# A Bead Biofilm Reactor for High-Throughput Growth and Translational Applications

**DOI:** 10.3390/microorganisms12081588

**Published:** 2024-08-05

**Authors:** Annika Gilmore, Marissa Badham, Winston Rudisin, Nicholas Ashton, Dustin Williams

**Affiliations:** 1 Department of Biomedical Engineering, University of Utah, Salt Lake City, UT 84112, USA; 2 Department of Orthopaedics, University of Utah, Salt Lake City, UT 84108, USAu1182343@utah.edu (W.R.);; 3 Department of Pathology, University of Utah, Salt Lake City, UT 84112, USA; 4 Department of Physical Medicine and Rehabilitation, Uniformed Services University of the Health Sciences, Bethesda, MD 20814, USA

**Keywords:** biofilm, reactor, repeatability, SEM, surface coverage, antibiofilm, in vitro, *S. aureus*, *P. aeruginosa*

## Abstract

Bacteria in natural ecosystems such as soil, dirt, or debris preferentially reside in the biofilm phenotype. When a traumatic injury, such as an open fracture, occurs, these naturally dwelling biofilms and accompanying foreign material can contaminate the injury site. Given their high tolerance of systemic levels of antibiotics that may be administered prophylactically, biofilms may contribute to difficult-to-treat infections. In most animal models, planktonic bacteria are used as initial inocula to cause infection, and this might not accurately mimic clinically relevant contamination and infection scenarios. Further, few approaches and systems utilize the same biofilm and accompanying substrate throughout the experimental continuum. In this study, we designed a unique reactor to grow bacterial biofilms on up to 50 silica beads that modeled environmental wound contaminants. The data obtained indicated that the reactor system repeatably produced mature *Staphylococcus aureus* and *Pseudomonas aeruginosa* biofilms on the silica beads, with an average of 5.53 and 6.21 log_10_ colony-forming units per mm^2^, respectively. The bead substrates are easily manipulable for in vitro or in vivo applications, thus improving translatability. Taken together, the bead biofilm reactor presented herein may be a useful system for repeatably growing established biofilms on silica beads that could be used for susceptibility testing and as initial inocula in future animal models of trauma-related injuries.

## 1. Introduction

Bacteria in nature preferentially reside in the biofilm phenotype [1,2]. Biofilms’ components, such as protective extracellular polymeric substances (EPSs), allow them to adhere to natural substrates like pebbles or sand particles [3,4]. The EPSs and other inherent characteristics of biofilms, including persister cells and antibiotic tolerance [5,6,7,8], make them risk factors for contaminated wounds such as open fractures [9], which are particularly problematic in military training and battlefield scenarios; biofilms dwelling on natural debris can contaminate damaged tissues at the point of injury. Although a civilian patient or soldier may receive systemic antibiotic therapy, their blood levels may be insufficient to effectively manage contaminating biofilms at wound sites. Furthermore, if their vasculature is compromised, systemic antibiotic therapy may not even reach the injured site [10]. This collective of problems contributes to high rates of difficult-to-treat infections [11]. In this study, we aimed to develop a reactor system in which biofilms can be grown on silica beads that more closely mimic biofilm-ridden sand or other particulate contaminants. Secondarily, we aimed to characterize the repeatability and reproducibility of this reactor. Ultimately, the goal of this work was to develop a system for growing biofilms on substrates that can be translated throughout the experimental continuum, ranging from in vitro susceptibility testing to being used as initial inocula in in vivo models of traumatic injuries and related infections.

Many biofilm reactor systems exist. All of them have pros and cons, depending on the experimental context, requirements, or hypotheses. The Centers for Disease Control (CDC) biofilm reactor from BioSurface Technologies (Bozeman, MT, USA) [12,13] is perhaps the most widely used reactor system for analyzing a myriad of experiments on robust, plumy biofilms [14,15,16,17]. The minimum biofilm eradication concentration (MBEC) Assay^®^ Kit from Innovotech (Edmonton, AB, Canada; formerly The Calgary Biofilm Device) is commonly used for susceptibility testing and imaging [18,19,20]. These systems are useful for in vitro analyses, but the substrates may be challenging to translate from in vitro to in vivo applications. In the case of an MBEC tray, the pegs on which biofilms are grown may not model a medical device or surface very closely if they were to be removed and implanted in an animal to model biofilm-related infections. 

One of our overall goals as a lab group is to develop systems that address these types of limitations. For example, in earlier studies, we used a rudimentary method to grow *Staphylococcus aureus* biofilms (n = 10) on silica beads in a Petri dish with broth exposed to mild rotation [21]. This approach was similar to that used by Konrat et al., who grew *Pseudomonas aeruginosa* biofilms on 3–5 mm diameter glass beads placed into the wells of a 24-well plate [22]. In our case, the beads modeled sand particles on which biofilms were present to more closely mimic environmental contaminants that affect traumatic injury sites. The biofilms were placed near traumatized bone and soft tissue and successfully exacerbated heterotopic ossification formation [21]. However, in later studies, we sought to develop a reactor that could produce biofilms on larger quantities of substrates for anti-biofilm screening. Further, we wanted to retain the same substrate type and reactor environment to allow the use of the same biofilm through anti-infective product development, from in vitro tests to in vivo models.

Here, we aimed to increase the substrate numbers and reduce biofilm growth challenges that were observed in the aforementioned study, including challenges such as when glass beads crashed into others in the Petri dish used and disrupted biofilms that had formed. To do so, we designed a system for retaining beads in a stationary tray within a crystallizing dish. We hypothesized that by standardizing the growth environment, mature biofilms would grow reproducibly on each glass bead.

## 2. Materials and Methods

### 2.1. Reactor Design and Prototyping 

**Figure 1:**
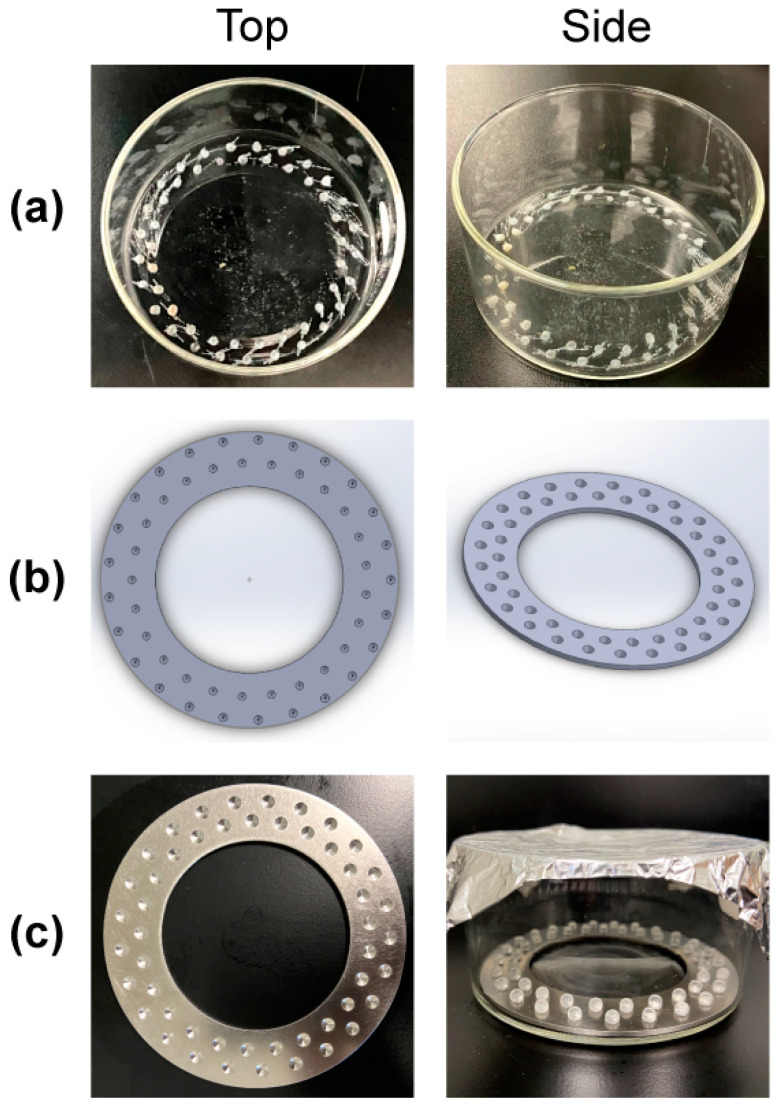
Reactor design from original prototype to final dish, viewed top-down and from the side. (**a**) Initial glass dish prototype with fifty divots etched with a Dremel tool. We found that this approach could adequately hold 4 mm glass beads in place when exposed to 40 rpm shear force. However, the manufacturing process was laborious and somewhat inconsistent. Biofilm growth could be seen adhering to Dremel surface defects along the dish. (**b**) A SolidWorks insert that contained fifty divots and could be exchanged between crystallizing dishes or manufactured with different materials. (**c**) The final reactor system with an aluminum foil lid. The metal insert prevented bead substrates from dislodging in the assembled reactor, even when broth was added in a biosafety cabinet.

An early prototype was created by dremeling fifty 3.5 mm divots in two concentric circles equidistant from the center of a Pyrex 100 mm × 50 mm (325 mL) crystallizing dish (Charleroi, PA, USA) (Figure 1a) to stabilize 4 mm glass bead substrates (sourced from Sigma Aldrich; St. Louis, MO, USA). Each divot had a depth of approximately 2 mm. However, the depths created using this manual method varied. Indeed, the variability from divot to divot was so notable and the manufacturing process was so time-consuming that we transitioned to an alternate approach and designed a circular ring insert (available as Supplementary File reactor_insert.SLDPRT) in SolidWorks (Dassault Systèmes SOLIDWORKS Corp; Waltham, MA, USA) (Figure 1b). The outer radius curve was 48 mm, and the inner one was 23 mm. Using CAD illustrations, the University of Utah’s medical machine shop machined our ring using 304 stainless-steel (Figure 1c). Stainless steel can be highly polished, preventing unwanted bacterial adhesion on the insert, and the stainless steel we used prevented rust formation following a bleach-based disinfecting protocol after reactor trials. 

The final reactor design (Figure 1c) comprised a Pyrex 100 × 50 mm crystallizing dish, 50 sanded glass beads, and a custom, polished 304 stainless-steel insert. Finally, we capped the reactor with an aluminum foil ‘lid’ and autoclaved the system at 121 °C for 30 min.

### 2.2. Reactor Substrate Preparation and Roughness 

To prepare substrates for biofilm growth, 10–15 silica beads were hand-sanded between two 60-grit pieces of sandpaper for 30 s. Bead substrates were cleaned by rinsing them in deionized (DI) water and drying them with a Kimwipe (Kimberly-Clark; Neenah, WI, USA). After cleaning, we examined their opaqueness. The diameters of beads were measured with calipers before and after sanding, but we did not detect a reduction. 

Confocal laser scanning microscopy (CLSM) (Olympus LEXT OLS 5000; Tokyo, Japan) was used to qualitatively assess the change in surface roughness between non-sanded and sanded bead substrates. Ten hand-sanded and ten non-sanded beads were randomly selected and imaged at 50×. This magnification negated the curvature of the bead, resulting in a more accurate assessment of surface roughness. We obtained the areal surface roughness of a 258 µm by 258 µm, or 66.5 mm^2^, area, using the arithmetical mean height (e.g., absolute value of height variations) across the entire 50× plane of view. From this, we calculated the average and standard deviation in terms of roughness across sanded and non-sanded beads.

### 2.3. Bacterial Isolates and Supplies 

Pathogens were chosen based on clinical and orthopedic trauma relevance [11,23,24]. *S. aureus* ATCC 6538 and *P. aeruginosa* ATCC 27853 were purchased from the American Type Culture Collection (ATCC) and maintained in frozen stocks (−80 °C) in brain heart infusion (BHI) broth with 30% glycerol. One to two days before use, isolates were subbed onto tryptic soy agar (TSA) plates and incubated at 37 °C overnight. To mimic an in vivo nutrient environment, brain heart infusion (BHI) broth from Research Products International (Mount Prospect, IL, USA) was used as the culture media. Ten × phosphate-buffered saline (PBS) from Thomas Scientific (Swedesboro, NJ, USA) was diluted to 1× with DI water before use. Both PBS and BHI broth were autoclaved at 121 °C with a holding time of 30 min prior to use.

### 2.4. Biofilm Growth 

Following autoclave sterilization of the reactor and bead substrates, glass beads were aseptically placed into each divot inside a biosafety cabinet. A 0.5 McFarland standard (~7.5 × 10^7^ colony forming units (CFU)/mL) was adjusted in PBS using one to three freshly subbed bacterial colonies. In total, 50 mL of 50% BHI broth and 1 mL of the 0.5 McFarland standard were aseptically added to the reactor to obtain an initial planktonic bacterial broth concentration of ~1.5 × 10^6^ CFU/mL. An aluminum lid was placed on the reactor (Figure 1c), and the reactor was placed in a rotating incubator set at 37 °C and 40 rpm for 72 h. These settings were selected to mimic the temperature and low-shear environment common to in vivo extremities. The bead reactor used was batch-styled and required manual exchanges of spent broth after 24 and 48 h. We replaced the spent broth with 50 mL of fresh BHI broth in a biosafety cabinet with a serological pipette.

### 2.5. Quantification and Statistical Analysis 

Biofilm beads were removed using sterile forceps and transferred individually to a tube containing 2 mL of PBS. The beads were vortexed for 1 min, sonicated for 10 min, and then vortexed again for 30 s and plated on TSA using a 10-fold dilution series. Plates were incubated overnight at 37 °C, and CFUs were counted the following day. Bioburden per mm^2^ was calculated using the average of countable CFUs from each dilution plate. A total of eight reactor trials were performed for *S. aureus* and *P. aeruginosa*, and six beads from each reactor were quantified for a total of 48 beads per organism. The bioburden per mm^2^ was log_10_-transformed, and the average and standard deviations were calculated between each reactor and run across eight trials. Data were analyzed using a linear mixed-effects model in Rstudio from Posit PBC (Boston, MA, USA). 

### 2.6. Fixation and Imaging

To image biofilms on beads using scanning electron microscopy (SEM), two to five beads were left in the reactor after each run, and modified Karnovsky’s fixative (2.5% glutaraldehyde and 4% formaldehyde in 0.2 M PBS) was gently added into the crystallizing dish until it covered the beads. Retaining substrates in the reactor helped reduce the movement and dislocation of biofilm during fixation and dehydration. Beads were fixed at room temperature for 24 h. Modified Karnovsky’s fixative was slowly removed using a serological pipette. Increasingly concentrated ethanol solutions (70%, 80%, 90%) were added for 1–3 h each to dehydrate samples. The beads were then left overnight in 100% ethanol and allowed to air-dry. Using forceps, fixed and dehydrated beads were placed onto a carbon-taped SEM stage and gold-coated using a Hummer 6.2 gold sputter coater (Anatech LTD, Battle Creek, MI, USA). In secondary electron imaging mode, beads were imaged with a JEOL JSM-6610 SEM (Tokyo, Japan).

To image biofilms on beads using fluorescence microscopy, four beads from each reactor run were removed and placed into a custom acrylic slide; the slide was 1 cm thick, with spherical divots drilled through the total thickness of the slide with decreasing diameters starting at 5 mm and ending at 3 mm. A glass coverslip was attached to the side with smaller diameter divots using superglue (3 M; Maplewood, MN, USA). The top portion of each bead was placed face down into the slide. Live/Dead stain (Thermofisher; Walton, MA, USA) was then applied, and samples were imaged using a Leica DMi8 inverted microscope via the THUNDER package (Wetzlar, Germany).

## 3. Results

General observation of the system and the outcomes obtained indicated that while the initial prototyped reactor (Figure 1a) adequately held the 4 mm diameter beads, creating the divots was laborious and introduced scratches that were inconsistent and could accommodate biofilm growth. The use of a metal insert (Figure 1b,c) resulted in a more homogeneous divot consistency, facilitated manufacturing, and mitigated biofilm growth on the crystallizing dish and insert surfaces. The sanded substrates had comparable *S. aureus* and *P. aeruginosa* bioburdens between beads of the same reactor and consistent growth between reactors. Fixation of biofilm to the curved glass surface was challenging, but it did reveal multilayered structures of both organisms.

### 3.1. Substrate Roughness Characterization 

**Figure 2:**
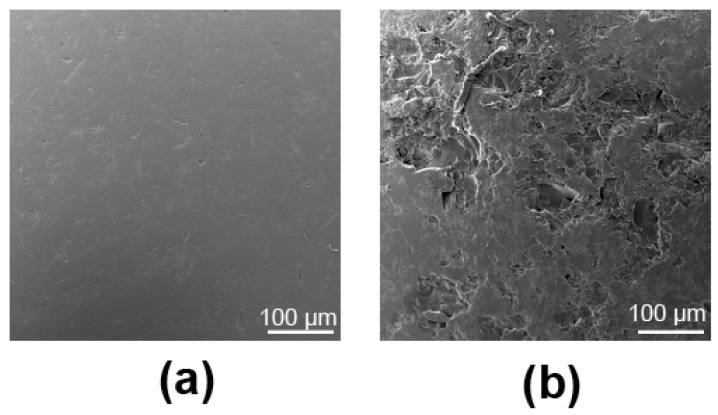
SEM images of a 4 mm glass bead surface before (**a**) and after (**b**) hand sanding it between two sheets of 60-grit sandpaper.

Sanding the beads with 60-grit sandpaper for 30 s created consistent yet notable roughness across the bead surface that could be seen with the naked eye. Roughness data showed that the mean arithmetic height of the non-sanded beads was 1.83 µm ± 0.32, and that of the sanded beads was 3.02 µm ± 0.83. The SEM images captured corroborated our visual observations. Specifically, while the surfaces of the non-sanded bead substrates showed minor defects (Figure 2a), the sanding procedure produced noticeably more ‘peaks’ and valleys across the glass surface than were originally present (Figure 2b). 

### 3.2. Biofilm Growth 

**Figure 3:**
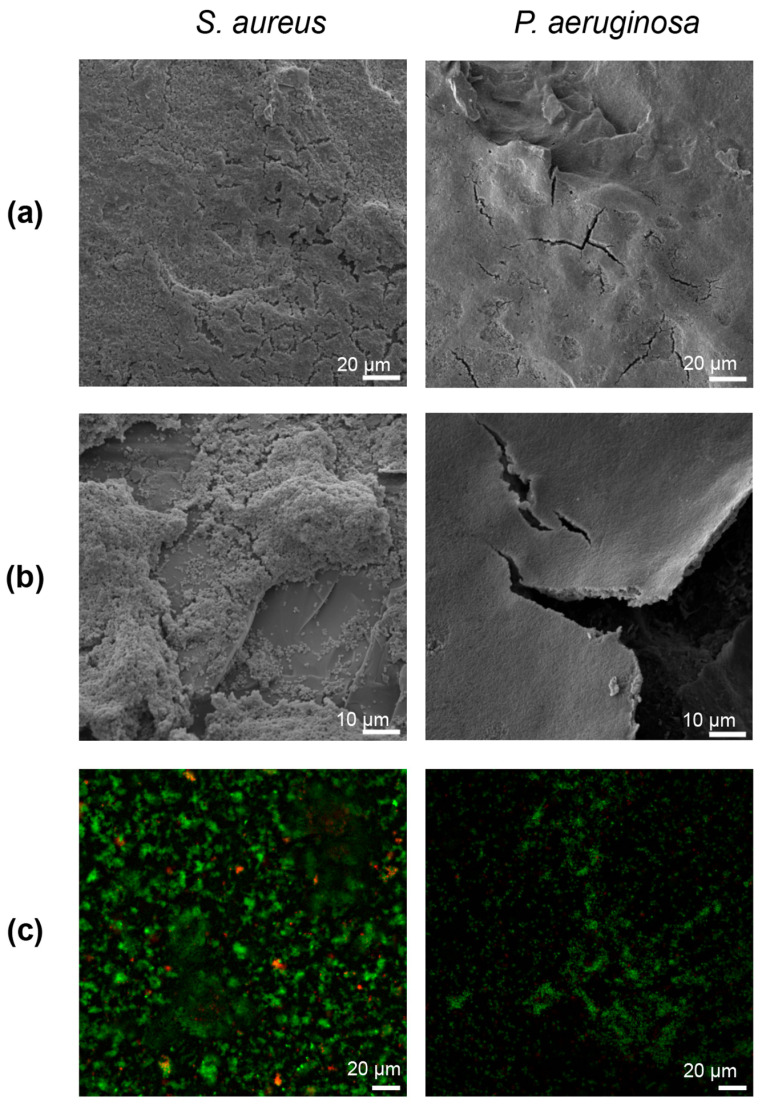
Images of *S. aureus* and *P. aeruginosa* biofilm growth on glass bead substrates at different SEM magnifications and live/dead-stained. (**a**) Representative SEM images of *S. aureus* and *P. aeruginosa* biofilm growth on the surface of a sanded glass bead taken at 500× magnification. *S. aureus* growth appeared to increase in thickness around textured areas, whereas *P. aeruginosa* dwelled in a multilayer sheet evenly covering the bead surface. (**b**) Representative SEM images of *S. aureus* and *P. aeruginosa* biofilm on the surface of a sanded glass bead at 1000× magnification. *S. aureus* growth correlated with substrate texture: biofilm plumes appeared to increase where sanded defects were most prominent. Comparatively, *P. aeruginosa* continued to grow in a sheet across the bead surface. (**c**) A representative z-slice image of live/dead-stained biofilms taken with an inverted light microscope showed predominately living (green) and fewer dead (red) stained bacteria cells. These images were taken at 60× magnification.

SEM images of the *S. aureus* and *P. aeruginosa* biofilm beads revealed copious amounts of biofilm formation across the roughened glass surfaces (Figure 3). *S. aureus* biofilm growth followed the bead surface geometry; where sanding created peaks or troughs, bacterial growth developed similarly (Figure 3b). Comparatively, *P. aeruginosa* biofilms coated the entire bead surface in a multicell layered sheet (Figure 3a,b). Fixation of *P. aeruginosa* to the glass beads with modified Karnovsky’s appeared to cause the biofilm/EPS to split away from the spherical substrate in some areas (Figure 3b). No apparent mushroom or pillar-like formations were present for either organism, morphologies that are commonly represented in biofilm images in the literature.

Inverted light microscope images of the live/dead stained biofilms revealed predominately green living cells (Figure 3c), suggesting that the growth process/reactor resulted in mature, highly viable biofilms.

### 3.3. Bacterial Quantification 

**Figure 4:**
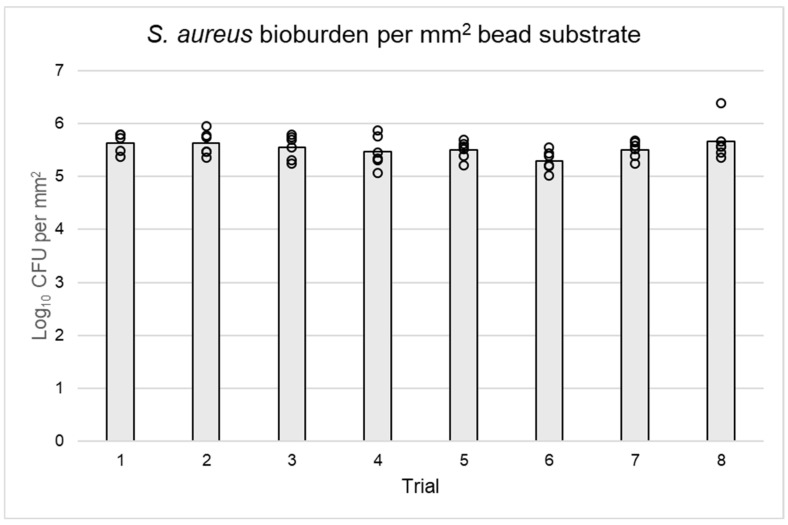
Log_10_ CFU per mm^2^ of *S. aureus* biofilm on beads grown in eight reactor runs. Gray bars represent the average CFU per eight trials, and black circles denote the specific CFU of each bead quantified (n = 6 per trial). There was greater variability in bioburden between beads of the same reactor than averages of each trial.

**Figure 5:**
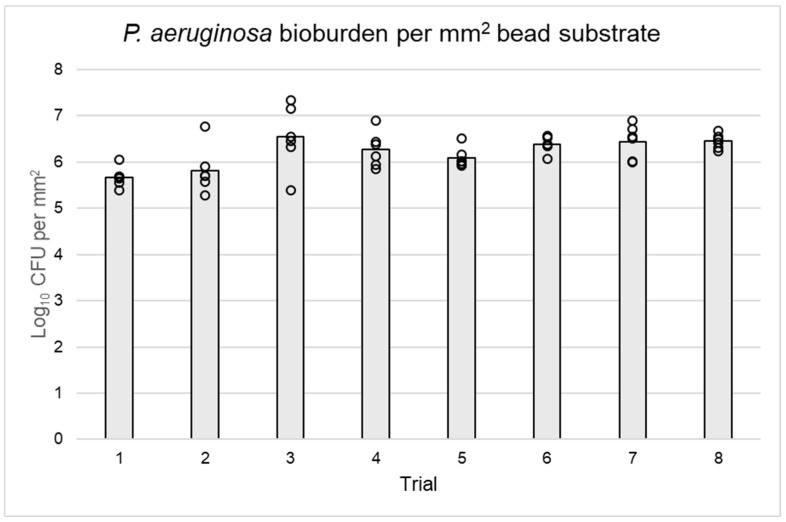
Log_10_ CFU per mm^2^ of *P. aeruginosa* biofilm on beads grown in eight reactor runs. Gray bars represent the average CFU per eight trials, and black circles the specific CFU of each bead (n = 6 per trial). Variability of *P. aeruginosa* bioburden occurred both between trials and within substrates of the same trial.

Uniformity of growth between and within reactor runs was confirmed (Table 1). The overall mean log_10_ densities were 5.53 log_10_ CFU/mm^2^ for *S. aureus* and 6.21 log_10_ CFU/mm^2^ for *P. aeruginosa*, with acceptable bioburden reproducibility between reactors and beads of the same trial (Table 1, Figure 4 and Figure 5). On average, *P. aeruginosa* biofilms on beads had at least 0.5 log_10_ CFU more bacteria per mm^2^ than *S. aureus*. The biofilm log_10_ densities exhibited acceptable reproducibility between substrates and trials, with more deviation occurring within runs than between them for both *S. aureus* and *P. aeruginosa* (Table 1). The original data used to make Table 1 and Figure 4 and Figure 5 are available as a Supplementary File.

In summary, our results show consistent substrate roughness, multi-layered *S. aureus* and *P. aeruginosa* biofilms that grew in a manner that followed bead topography, and consistent CFU counts within and between reactor runs with acceptable variance.

## 4. Discussion

There is no single biofilm reactor that addresses every experimental or translational need. The motivation to develop the bead biofilm reactor discussed herein stemmed from a need to grow biofilms on environmentally relevant substrates under more standardized conditions. Secondarily, there is a paucity of reactors that can grow biofilms on substrates that can be translated from benchtop to in vivo applications. And many reactor systems have very-high-volume broth requirements, which can be expensive and challenging to work with. As the data show, the bead biofilm reactor addressed these limitations and supported our hypothesis. 

The repeatability and reliability of the reactor supported our objective, producing *S. aureus* and *P. aeruginosa* biofilm counts that were within acceptable limits. Yet the slight intra-reactor variability of *P. aeruginosa* may have been a result of thicker EPS formation on some substrates than on others, which may have been underpinned by the number of cells dividing in a given microregion or disruption from handling with forceps.

Given the repeatable outcomes, one of the benefits of the prototype reactor is the potential for multiple substrate types to be used. Further work will be needed to determine how well or the degree to which biofilms form on alternate substrates within the reactor and how those substrates can be integrated into experimental protocols. If materials are used, they should be negatively buoyant as there are no mechanisms other than gravity to hold growth substrates in place. We chose to use silica (glass) because it is a prominent material used in biofilm testing [25] and mimics sand particles that commonly contaminate trauma sites. Silica is also inexpensive, easy to find, and easy to disinfect. Further, we can translate glass substrate inocula for in vivo work [21], and glass may be representative of other biomedical implants [26].

Previously, we and other researchers studied how surface roughness and the substrate used impact *S. aureus* and *P. aeruginosa* biofilm growth [14,27,28] and found that roughness increases the adherence of bacterial cells. Thus, standardizing a way to generate roughness in this study was beneficial. Overall, hand sanding was simple and allowed for frequent opacity checks to determine if more sanding was needed. 

However, a notable drawback of using silica was the challenge of fixation for SEM imaging. For instance, sheets of *P. aeruginosa* consistently flaked or tore away from rounded substrates. Using modified Karnovsky’s and retaining beads in the reactor for fixation and dehydration reduced flaking. Slow aspiration of chemicals further reduced disruption and preserved biofilm structures.

As for the comparison of bioburdens, the bead reactor seemed to produce biofilm quantities that were more similar to a CDC biofilm reactor than those grown on pegs in an MBEC system. CDC reactors produce mature biofilms, with thicker multi-layered structures; researchers have reported values between 6.10 and 6.30 log_10_ CFU/mm^2^ for *S. aureus* and 6.23 and 6.5 log_10_ CFU/mm^2^ for *P. aeruginosa* biofilms grown on glass coupons [29,30]. Biofilms grown on MBEC pegs are typically less robust, with some researchers reporting single-layered bacterial cell adhesion with an average of 3.83 log_10_ CFU/mm^2^ for *S. aureus* and 4.83 log_10_ CFU/mm^2^ for *P. aeruginosa* [31,32]. A cursory comparison of the bead reactor to established CDC and MBEC reactors is shown in Table 2. These comparisons are important as biofilm tolerance of antimicrobials is known to be dependent on the aerial cell density and age of a biofilm [33]. Future research will compare the antibiotic tolerance profiles of biofilms grown in various types of reactors.

A benefit of the bead reactor is its relatively small size. The bead reactor grows biofilm within a rotating incubator, reducing variance that might result from changes in ambient room temperature. Comparatively, the CDC biofilm reactor often requires insulating materials around the reactor casing to maintain temperature; the CDC reactor is heated via a hotplate positioned within a room-temperature lab. Furthermore, the bead reactor can be moved into a closed container to model anaerobic growth. And because of the reactor’s low volume, minimal broth is required, which might promote future use of more expensive growth media, such as artificial sputum. 

Another aspect of this reactor system, which may not be a limitation depending on experimental requirements, was that it required low shear force. In early experiments, we rotated the reactor at 100 rpm, which caused silica beads to fall out of divots. Without securing beads in some way, growth is limited to lower shear (i.e., rpm). The stainless-steel dish insert can hold glass beads in place at up to 45 rpm. Alternatively, lower shear could also be used. We did not quantify the resulting bioburden from different shear forces but opted for 40 rpm to mimic conditions relevant to environmental debris.

When growing and testing biofilm, the conditions in which naturally occurring biofilm dwell should be considered [29,34]. On the battlefield, debris often enters wound sites in the form of sand, dirt, dust, or clothing. Biofilms that adhere to such substrates are unlikely to be exposed to a high-shear, nutrient-rich environment. Thus, when producing biofilms to model orthopedic trauma, low-shear (40 rpm), batch-phase growth may be more appropriate.

## 5. Conclusions

Taken together, we developed a bead biofilm reactor in which robust biofilms form on glass substrates with an average of log_10_ 5.53 CFU/mm^2^ of *S. aureus* and 6.21 CFU/mm^2^ of *P. aeruginosa*. The use of a dish reactor allowed biofilm growth on a greater number of substrates, which can be translated to numerous in vitro and in vivo applications, facilitating the use of the same biofilm and substrate across the continuum of anti-infectious technologies. Current studies are being conducted to assess biofilm susceptibility profiles between reactor types, and using biofilm-ridden silica beads in animal models of trauma-related infection. Future work is also considering mimicking other conditions (e.g., anaerobic growth) and alternative biomaterial substrates.

## Figures and Tables

**Table 1:** Statistical outcomes including average bioburden per mm^2^ of *S. aureus* and *P. aeruginosa* growth between beads of the same reactor and between reactor runs. The bead reactor produced comparable bioburdens per substrate within and across trials.

Organism	Mean Log_10_ CFU/mm^2^	SE ^1^	Repeatability SD ^2^	% Contribution between/within Runs
*S. aureus*	5.53	0.041	0.062	6.30/93.70
*P. aeruginosa*	6.21	0.11	0.23	33.96/66.04

^1^ Standard error. ^2^ Standard deviation.

**Table 2:** Average bioburden of *S. aureus* and *P. aeruginosa* between standard MBEC and CDC reactor growth methods in which glass substrates were used. Mean log_10_ CFU/mm^2^ of MBEC and CDC biofilm was obtained by exponentiating reported outcomes, recalculating them in CFU per mm^2^, and then log-transforming them. Reporting outcomes per substrate area facilities a direct comparison between biofilms on smaller surfaces areas, such as MBEC pegs and glass beads, and biofilm on larger CDC coupons.

Organism	Biofilm Structure	*S. aureus* Mean Log_10_ CFU/mm^2^	*P. aeruginosa* Mean Log_10_ CFU/mm^2^	References
MBEC Peg	Single-layered	3.83	4.83	[31,32]
Glass bead	Multilayered	5.53	6.21	
Glass coupon	Multilayered	6.10–6.30	6.23–6.5	[29,30]

## Data Availability

The original contributions presented in this study are included in the article/Supplementary Materials; further inquiries can be directed to the corresponding author.

## Supplementary Materials

The following supporting information can be downloaded at https://www.mdpi.com/article/10.3390/microorganisms12081588/s1. TBD: Full SEM images (Figure 2 and Figure 3); Table 1, Figure 4 and Figure 5, bioburden data; and an STL file for the ring insert.
