# Supplementary figures and images for: A Bead Biofilm Reactor for High-Throughput Growth and Translational Applications
Source: Microorganisms. 2024 Aug 5;12(8):1588. doi: 10.3390/microorganisms12081588 (PMC11356137; doi:10.3390/microorganisms12081588)

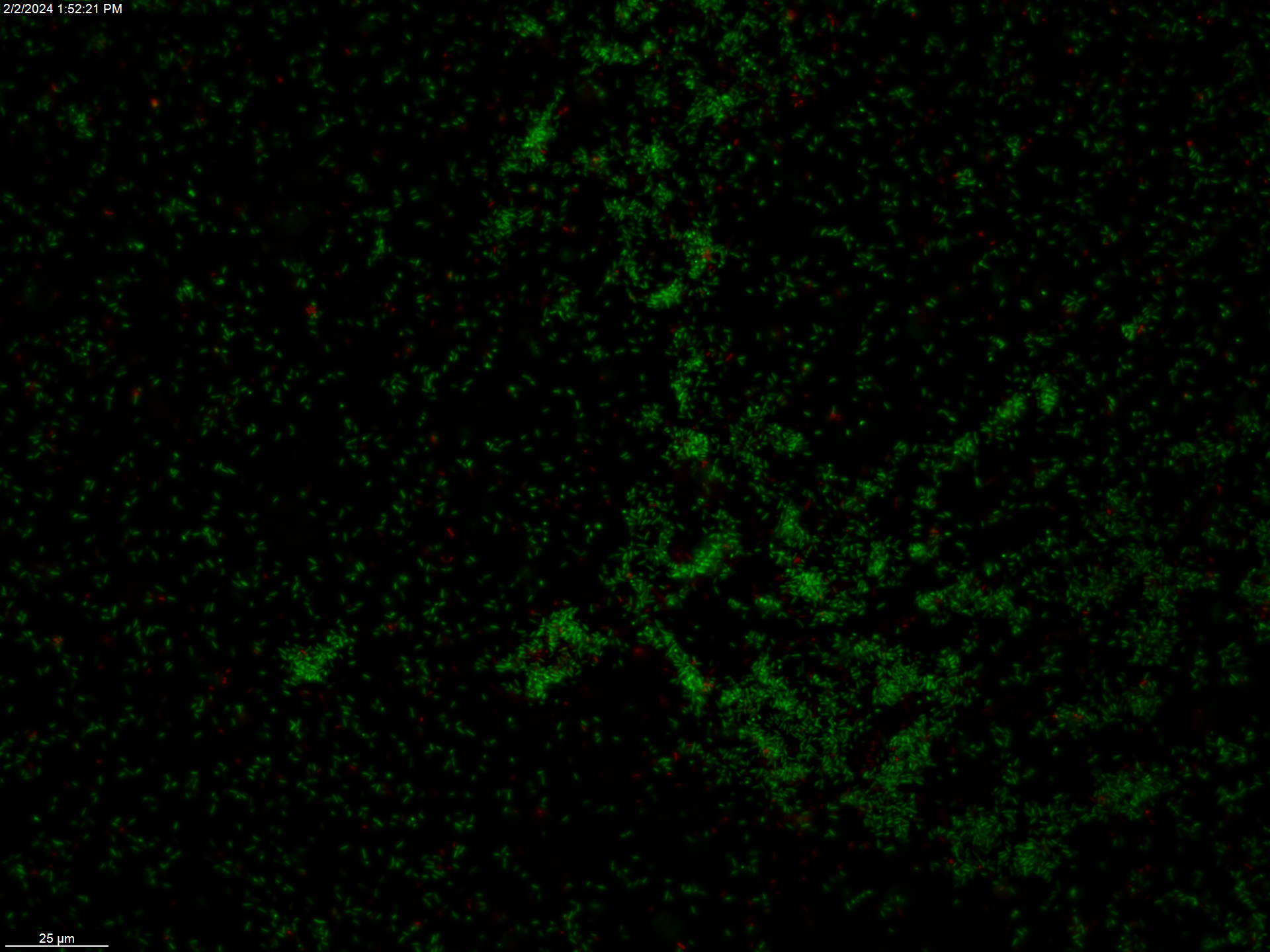

Supplement: Supplementary file 1 [file microorganisms-12-01588-s001.zip › TBD/original_figure S3c_pseudo.tif]

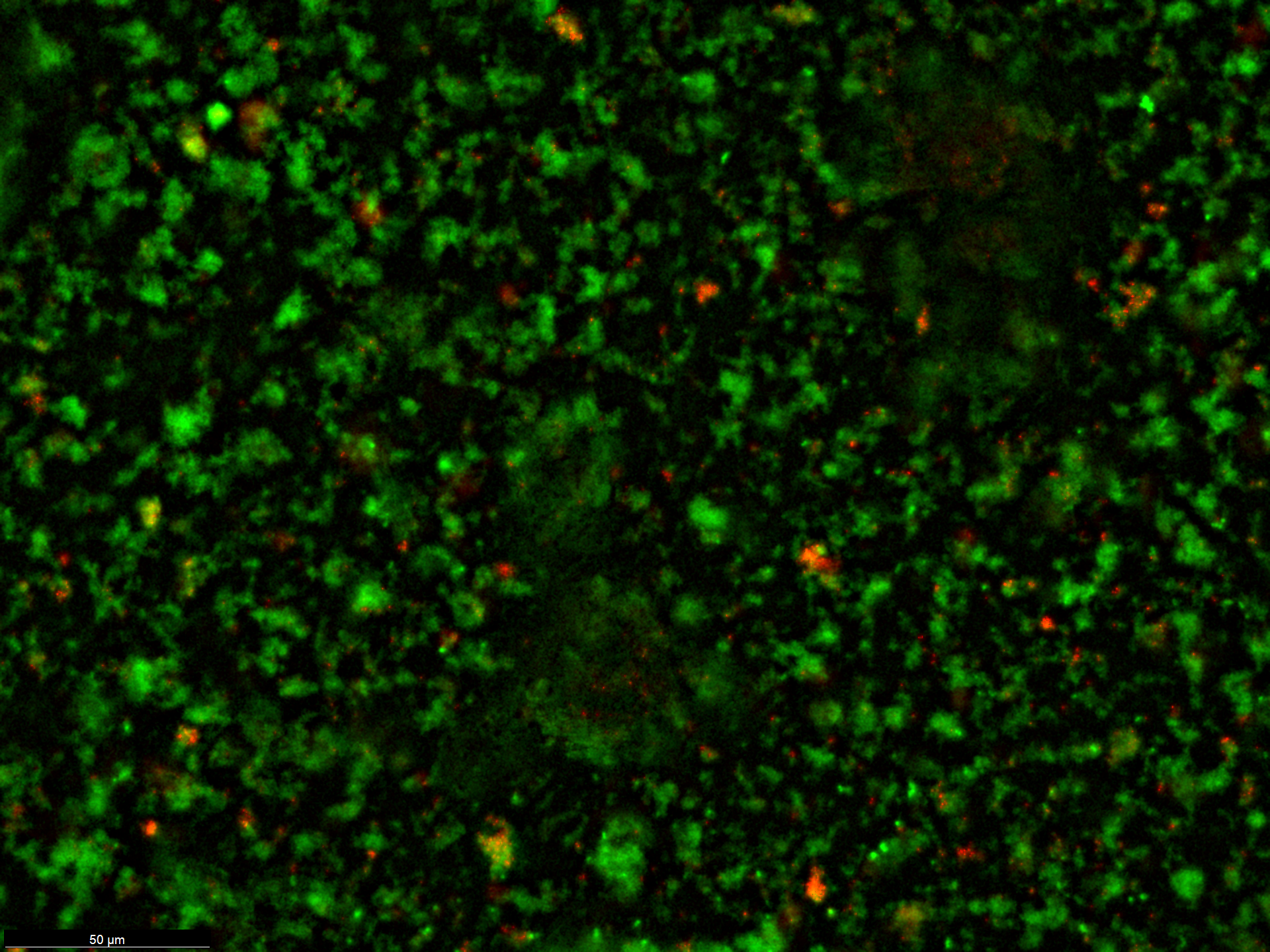

Supplement: Supplementary file 1 [file microorganisms-12-01588-s001.zip › TBD/original_figure S3c_staph.tif]
